# A randomised clinical trial of a metaphylactic treatment with tildipirosin for bovine respiratory disease in veal calves

**DOI:** 10.1186/s12917-017-1097-1

**Published:** 2017-06-14

**Authors:** J. Berman, D. Francoz, J. Dubuc, S. Buczinski

**Affiliations:** Département des sciences cliniques, Faculté de médecine vétérinaire, Université de Montréal, Saint-Hyacinthe, QC J2S 7C6 Canada

**Keywords:** Tildipirosin, Lung consolidation, Pneumonia, Ultrasonography, Weight gain, Bronchopneumonia

## Abstract

**Background:**

Bovine respiratory disease (BRD) is a major problem in veal calf rearing units. The objective of this randomised clinical trial was to assess the effectiveness of tildipirosin as a metaphylactic treatment in veal calves on the number of BRD treatments, lung consolidation on thoracic ultrasonography (TUS) and average daily gain (ADG). A total of 209 veal calves from a pre-weaning fattening unit were randomly allocated to receive one of two treatments (tildipirosin 4 mg/kg, subcutaneously, *n* = 109; placebo 0.9% saline, subcutaneously, *n* = 100) at day 12 after entry in the pre-weaned unit. The calves were followed for a 70-day period. Occurrence of mortality and BRD treatments were recorded during the pre-weaning period. At days 1, 12 and 30, TUS and clinical scores were performed and ADG was measured during the first and second months of feeding.

**Results:**

The use of a metaphylactic treatment of tildipirosin 12 days after arrival of the veal calves was not associated with the number of BRD treatments performed by the producer, ultrasonographic lung consolidation or weight gain (*P* < 0,05). In this cohort of calves, the proportion of calves treated for BRD by the producer was low at 14% (29/209). However, 13% (26/209) of calves included in the study already had ultrasonographic lung consolidation lesions 12 days after their arrival, which was before treatment time, and 27% (56/209) had lung consolidation at day 30.

**Conclusion:**

In this study population with a low BRD prevalence, we were not able to detect any benefit of tildipirosin as a metaphylactic treatment of BRD at day 12 after arrival based on BRD treatments, TUS, and ADG.

**Electronic supplementary material:**

The online version of this article (doi:10.1186/s12917-017-1097-1) contains supplementary material, which is available to authorized users.

## Background

Bovine respiratory disease (BRD) is a multifactorial disease involving an interaction between stressors, the environment, viruses and bacteria. BRD remains a major disease in all types of bovine production systems especially in veal calf production due to stress factors such as transportation, commingling from different sources, high prevalence of failure of passive transfer and veal calf processing [1]. In this production the mortality rate of BRD is low varying from 1.5% to 2% [1, 2], but the morbidity rate could be important according to the cohorts varying from 7% to 61% [1–3]. At slaughter, prevalence of calves with lung lesions is much higher and varies from 41% to 70% [4, 5]. Discrepancy between the clinical prevalence of BRD (i.e. the proportion of calves treated individually by producers or clinical cases) and the prevalence of lesions at slaughter indicates that many cases remain subclinical and undetected, contributing to long term losses particularly with individual treatment strategies [5].

Consequently, metaphylactic antimicrobial therapy is commonly used in the veal calf industry [6]. Metaphylactic antimicrobial therapy has been defined by Nickell and White as a mass treatment of animal populations currently experiencing any level of disease before the onset of blatant illness [6, 7]. The administration of metaphylactic treatment is usually instituted before the main peak of BRD clinical incidence [6] that occurs about three weeks after arrival at the fattening unit [1]. In Europe, metaphylactic antimicrobial treatments in veal calves are mostly administered by the oral route. By this route, the majority of cases, the antimicrobial drug used is underdosed compared to the labelled dose essentially because of underestimation of weight [8]. This raises concerns about the effectiveness of oral metaphylactic antimicrobial treatments and the development of antimicrobial resistance [8, 9]. In order to reduce the use of antimicrobial drugs and resistance development, parenteral administration of targeted antimicrobial drugs is reported as an alternative [9]. In veal calves, tulathromycin is the only antimicrobial drug for which a study on the effectiveness of its parenteral administration has been published [9]. No differences in growth performance or clinical signs compared to oral treatment with tetracycline were found, but a difference between the treated control group and the placebo was observed [9]. Tildipirosin (Zuprevo; Merck Animal Health, Kirkland, Québec, Canada), is another macrolide antimicrobial drug rapidly and extensively distributed to the respiratory tract followed by slow elimination [10]. In vitro, Tildipirosin is reported to be efficacious against *Pasteurella multocida, Mannheimia haemolytica* and *Histophilus somni* [11]*.* Its effectiveness against *Mycoplasma bovis* in vitro is reported but the CMI are higher than those for the others agents [12]. Tildipirosin is already approved for treatment and control of BRD in cattle in different countries. However, to our knowledge, the effectiveness of tildipirosin metaphylactic treatment, including its effect on growth performance in high-BRD risk calves, has never been studied in veal calves.

The main objective of this study was to assess the effectiveness of tildipirosin as a metaphylactic antimicrobial treatment on the average daily gain (ADG) in veal calves. Secondary objectives were to evaluate the effect of this metaphylactic treatment on the number of calves treated for BRD by the producer during the pre-weaning period and on the number of calves with lung consolidation on thoracic ultrasonography (TUS). The first hypothesis was that treated calves would have a greater ADG than non-treated calves. The second hypothesis was that calves treated with tildipirosin two weeks after arrival would have fewer BRD treatments and fewer ultrasonographic lung lesions than non-treated calves.

## Methods

A double-blinded randomised clinical trial was conducted between the months of July and October of 2014. The experimental protocol was approved by the Comité d’éthique et d’utilisation des animaux de l’Université de Montréal (protocol: 14-Rech_1727).

### Population and study design

The study was performed in one commercial pre-weaning veal calf fattening unit in St-Hélène de Bagot, Québec (Délimax, Veaux Lourds Ltée, Saint-Hyacinthe, Québec, Canada). Calves coming from local auction markets were enrolled at the approximate age of seven days of life (exact age was not reported for each calf) on arrival at the farm. A sample size estimation of 200 calves (100 calves per treatment group) was based on detecting a difference in treated versus control calves of 4 kg of weight at the end of the pre-weaning period (105 kg in control vs 109 kg in treated calves; assuming a population variance (s^2^) of 100) as well as a difference of 15% in the proportion of calves treated for BRD (25% in control vs 10% or less in treated calves) with a statistical power of 80% and an alpha error of 5%. The difference of 4 kg was defined as the minimal difference financially beneficial for the producer considering the cost of tildipirosin. The proportions used in this study were estimated based on previous data of BRD reported in veal calves by the owner and the literature [1], and the differences were based on those observed in feedlots [11].

On arrival at the farm (day 0, D0), calves were immediately intra-nasally vaccinated against infectious bovine rhinotracheitis and para-influenza 3 virus (Nasalgen® IP, Merck Animal Health, Kirkland, Québec, Canada) and were housed in duckboard individual pens and fed with milk replacer (21% crude protein, 18.5% fat) until a quantity of 25 to 28 kg at the time of weaning period 70 days after arrival. Each calf was identified using an ear tag, and calf pen allocation was also recorded. Calves were randomly distributed between two groups using the RAND function in Excel (Microsoft, Redmond, WA, USA) software. One group of calves received tildipirosin (TILD) at the labelled dose subcutaneously (SQ) (4 mg/kg). The second group received an equivalent volume of 0.9% saline SQ (PLAC). The metaphylactic antimicrobial and placebo treatments were given at day 12 (D12), which was one week before the main peak of clinical incidence according to historical health observations in this fattening unit (Annie Dubuc, Dr. Frédéric Beaulac, Délimax veaux lourds Ltée, personal communication) that is similar of the peak reported in veal calves in the litterature [1].

The commercial solution of tildipirosin (18%) and sterile saline (0.9%) were specifically prepared for the trial, using opaque vials, and they were kept locked away by a pharmacist. The bottles were labelled with either an A or a B, and the corresponding keys were kept by the pharmacist during the entire study period. The bottle identifications were only revealed to the researcher at the end of the data collection period.

### Data collection

**Fig. 1:**
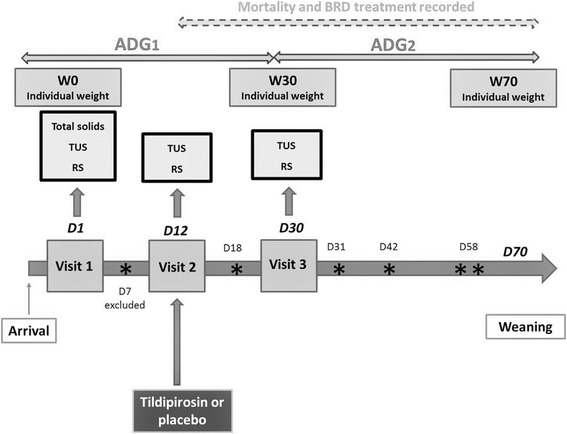
Timeframe of the study period with the different data collected and their times of collection as well as calf deaths during the study. Legends. D: Day; W: Weight; TUS: Systematic thoracic ultrasonography; RS: Respiratory score; ADG1: Average daily gain for the first month of feeding; ADG2: Average daily gain for the second month of feeding; (*) represents dead calves; *dotted arrow* represents the mortality and BRD treatment recorded and integrated in the statistical analysis

The different data collected and their times of collection are presented in Fig. 1.

The first visit was performed by a veterinarian and an animal health technician one day after arrival (D1). Calves were bled from the jugular vein for assessment of serum total solids. Blood samples were centrifuged at 3500 rpm for 10 min at room temperature. The serum total solids were then determined using a manual handheld refractometer (Atago SUR-NE, Tokyo, Japan). Failure of passive transfer (FPT) was defined when total solids were less than or equal to 52 g/l [13].

Throughout the study period, the producer was allowed to treat the calves according to on-farm protocols but did not participate in the research project visits. Individual medical treatments or events, with a focus on BRD treatments, and mortalities were recorded during the entire pre-weaning period but no necropsies were performed. The number of BRD treatments was used to estimate the apparent prevalence by the producer.

On D1, D12 (the day of metaphylactic treatment) and day 30 (D30), a respiratory score (RS) was also performed on each calf using the Calf Respiratory Scoring Chart [14] as previously described [15]. An RS of five or more has been suggested as a BRD clinical case [14]. All clinical examinations were performed by two different operators blinded to TUS results and treatment allocation.

On D1, D12 and D30, TUS was performed with a 7.5 MHz linear probe (Imago, ECM, Angoulème, France) on the right and left thorax by two different operators blinded to respiratory score results and treatment allocation. The area screened was the mid to ventral portion of the right and left lung as well as the parenchyma cranial to the heart [16]. Thoracic ultrasonography was used to detect calves with lung consolidation. The maximal depth of consolidation (DEPTH) was recorded. The DEPTH (cm) was calculated by a manual count of the 1 cm squares using the 1 cm^2^ grid of the ultrasound unit. As previously described, lung consolidation was considered significant when at least one point of DEPTH greater than or equal to 3 cm was observed [16].

During the study period, the calves were weighed by a blind research assistant three times: at arrival (D0, W0), at D30 (W2) and just before leaving the fattening unit at day 70 (W3). Individual calf weights were determined by a digital scale. Average daily gain was defined for the first month of feeding (ADG1; difference between W2 and W0 divided by 30 days), for the second month of feeding (ADG2; difference between W3 and W2 divided by 40 days) and for the total of the pre-weaning period (ADG tot; difference between W3 and W0 divided by 70 days).

### Statistical analyses

The calf was the unit of interest in this study. As recommended by Sargeant [17], intention-to-treat (ITT) analyses were used. This analysis compares the group exactly as randomised. This approach is considered the gold standard if there are losses to follow-up in a randomised controlled trial because randomisation is maintained and adverse effects, as well as lack of compliance, is likely to occur in “real world” use of the intervention [17]. For the only calf that died before the third visit at D30, the medians of ultrasonographic lung consolidation, of clinical score and of ADG, were assigned [18]. For the calves that died after D30, a last observation carried forward approach [18] was performed for ADG, i.e. the ADG during the second period of the dead calves (ADG2) was considered similar to the one observed during the first period (ADG1; ADG1 = ADG2). Two calves had no weight at D30 to calculate ADG1 and ADG2, and ADG tot was attributed to calves.

Statistical analyses were performed using SAS (version 9.3, SAS Institute Inc. Cary, NC, USA). Descriptive statistics were first calculated for the two treatment groups using the MEANS and FREQ procedures in SAS. A T-test was used to compare both treatment groups concerning the continuous variables (total solids at arrival, weight, ADG), with statistical significance set at *P* < 0.05. Fisher’s exact test was used to compare both treatment groups concerning the dichotomized variables (breed, sex, FPT, TUS, RS, mortality, BRD treatments by the producer after administration of TILD), with statistical significance set at *P* < 0.05.

Secondly, logistic regression analyses were performed to assess the impact of treatment group on the presence of significant lung consolidation on D30 (GLIMMIX procedure with LOGIT link in SAS). Univariable model building was performed using Chi-squared or Fisher’s exact tests (FREQ procedure). The covariates with *P* < 0.25 were retained for building a final multivariable linear regression model using a backward elimination strategy. The final model was obtained when all covariates had *P* < 0.05.

Finally, ADG1, ADG2 and ADG tot were considered as dependent variables in univariable linear regression models (MIXED procedure in SAS). All the potential covariates were first assessed using univariable analyses with ADG as the dependent variable. The covariates with *P* < 0.25 were retained for building a final multivariable linear regression model using a backward elimination strategy. The final model was obtained when all covariates had *P* < 0.05. The treatment group was forced in every multivariable model since it was the main study interest.

## Results

Two hundred and ten dairy calves, with an average weight of 51.7 kg (SD = 6.4 kg) were enrolled in the study. One calf died at day seven, before metaphylactic treatment, and was therefore excluded from the study. At D12, a total of 209 calves received either tildipirosin (TILD, *n* = 109) or the placebo (PLAC, *n* = 100). No adverse drug effect was observed.

Descriptive statistics at arrival (D1) and at the moment of treatment administration (D12) are presented in Table 1. Five calves died during the whole pre-weaning period. The times of death during the study period are described in Fig. 1. All calves who died during the study were in the PLAC group (Fisher’s exact test; *P* = 0.02). Among the dead calves, four had lung consolidation with DEPTH > 3 cm at the last TUS before death, and two among this four had been treated for BRD. No differences were observed for the number of calves treated for BRD during the entire pre-weaning period (TILD group: 17/109 (16%) vs PLAC group 18/100 (18%) (Fisher’s exact test; *P* = 0.64). Between D12 and D30, three antimicrobial treatments for another indication of BRD were used. They were not included in the statistical analysis.

**Table 1:** Descriptive statistics at days 1 (D1) and 12 (D12) of the 209 calves randomly assigned to receive a metaphylactic antimicrobial treatment of tildipirosin (TILD) or placebo (PLAC) at D12

	PLAC (*n* = 100)		TILD (*n* = 109)	
Nominal variables*	Number	Proportion (%)	Number	Proportion (%)
Male	91	91	103	95
Holstein breed	89	89	99	93
RS ≥ 5^a^				
D1	1	1	0	0
D12	3	3	5	5
DEPTH ≥ 3 cm^b^				
D1	1	1	2	2
D12**	8	8	18	17
Failure of passive transfer^c^	55	55	59	54
Continuous variables*	Mean (range)	SD	Mean (range)	SD
Serum total solids (g/L)	54.3 (38–83)	7.9	53.3 (41–80)	7.9
Weight at arrival (kg)	47.6 (34.5–63.5)	5.9	46.7 (33.1–65.3)	6.8

^a^Calf respiratory score assessing rectal temperature, cough, nasal and ocular discharge as well as ear drop. Calf is considered clinically sick when score ≥ 5

^b^Ultrasonographic assessment of lung consolidation maximal depth using thoracic ultrasonography. Calf is considered sick by ultrasonography when depth ≥ 3

^c^Serum total solids less than 52 g/l

*Used of Fisher’s test or chi-squared test for the nominal variables and t-test for the continuous variables

**P = 0.06; no significant difference observed for all data

At D30, 29 (27%) calves had ultrasonographic lung consolidation in the PLAC group versus 27 (27%) calves in the TILD group (Chi-squared test; *P* = 0.91). Among these 56 calves, 14 already had ultrasonographic lung consolidation at D12 (9 in PLAC group and 5 in TILD group). Data concerning the proportion of subclinical and clinical calves are presented in Additional file 1: Table S1. In the univariable analyses, the treatment group was not associated with the presence of ultrasonographic lung consolidation at D30 (*P* = 0.89). The final multivariable model showed that, when accounting for treatment group (TILD vs PLAC), ultrasonographic lung consolidation at D30 was not different between groups (*P* = 0.78). Presence of lung consolidation at D12 and FPT were associated with the presence of consolidation at D30 in this model (*P* < 0.05; Table 2).

**Table 2:** Results from the multivariable logistic regression analysis showing variables that were associated with the presence of lung consolidation at D30

Variables		Coefficient	SE	OR	95% CI	*P*-value
Treatment	PLAC	Referent	-	-	-	-
	TILD	−0.09	0.33	0.91	0.47–1.76	0.78
DEPTH d12	<3 cm	Referent	-	-	-	-
	≥3 cm	1.47	0.45	4.35	1.79–10.6	<0.01
Total solids	>52 g/L	Referent	-	-	-	-
	≤52 g/L	0.85	0.34	2.35	1.19–4.65	0.02

*PLAC* placebo treated calves, *TILD* tildipirosin treated calves, *DEPTH D12* maximal depth of lung consolidation found during thoracic ultrasonography at day 12 after arrival (i.e. time of injection of TILD or PLAC).

Results of the univariable analyses reported no association between ADG1 and potential covariates (*P* ≥ 0.25). Lung consolidations at D30 and BRD treatments by producer were both associated with ADG2 and with ADG tot (*P* < 0.25; Table 3). The final multivariable model showed that, after adjusting for treatment groups (TILD vs PLAC), BRD treatments by producer (*P* < 0.01) was negatively associated with ADG2 and ADG tot. The effect of the metaphylactic treatment was not significant (*P* = 0.07; Table 4).

**Table 3:** Results of the univariable analyses assessing the association between the average daily gain during the 1^st^ month of feeding (ADG1), the 2^nd^ month of feeding (ADG2) and during whole the pre-weaning period (ADG tot), and potential covariates measured during the study

		*ADG 1*			*ADG 2*			*ADG tot*		
Variables		LSM (lbs/d)	SEM	*P-*value	LSM (lbs/d)	SEM	*P-*value	LSM (lbs/d)	SEM	*P-*value
Treatment	PLAC	1.32	0.05	0.37	2.22	0.05	0.37	1.90	0.04	0.97
	TILD	1.28	0.05		2.27	0.04		1.90	0.04	
Sex	Female	1.42	0.13	0.33	2.19	0.12	0.68	1.91	0.10	0.95
	Male	1.29	0.04		2.24	0.03		1.90	0.03	
DEPTH D1	≥ 3 cm	1.39	0.28	0.71	2.55	0.26	0.25	2.13	0.23	0.35
	< 3 cm	1.31	0.03		2.26	0.03		1.92	0.03	
DEPTH D12	≥ 3 cm	1.17	0.10	0.25	2.30	0.09	0.58	1.89	0.08	0.78
	< 3 cm	1.29	0.04		2.26	0.03		1.91	0.03	
DEPTH D30	≥ 3 cm	1.28	0.07	0.93	2.34	0.06	0.13	1.97	0.05	0.17
	< 3 cm	1.29	0.04		2.24	0.04		1.89	0.08	
Total solids	≤ 52 g/l	1.29	0.05	0.95	2.25	0.04	0.98	1.91	0.04	0.83
	>52 g/l	1.30	0.05		2.25	0.05		1.90	0.04	
BRD treatments	0	NA			2.29	0.03	< 0.01	1.95	0.03	< 0.01
	≥ 1	NA			1.85	0.15		1.42	0.22	

*LSM* Least Squares Means, *SEM* Standard Error of the Mean, *DEPTH D1* maximal depth of lung consolidation found during thoracic ultrasonography at arrival, *DEPTH D30* maximal depth of lung consolidation found during thoracic ultrasonography at day 30 after arrival. BRD treatments represent the proportion of calves treated for bovine respiratory disease by the producer during the feeding period. NA: not available. The breed (not presented here) was not significantly associated with ADG1 (*P* = 0.30) or ADG2 (*P* = 0.26).

**Table 4:** Results of the multivariable linear regression analysis showing variables that have an impact on average daily gain during the second month of feeding (ADG 2)

Variables		Estimate	SE	*P*-value	LSM (lbs/d)	SEM
	Intercept	1.7305	0.19	<0.0001		
Treatment	PLAC	Referent			2.06	0.08
	TILD	0.12	0.07	0.07	1.94	0.08
DEPTH D30	<3 cm	Referent			1.97	0.07
	≥3 cm	−0.07	0.09	0.47	2.05	0.09
BRD treatments	0	Referent	0.18	<0.01	2.29	0.05
	≥1	−0.41	0.25		1.87	0.16

*LSM* Least Squares Means, *SEM* Standard Error of the Mean, BRD treatments represent the proportion of calves treated for bovine respiratory disease by the producer during the feeding period.

## Discussion

The use of a metaphylactic treatment of TILD 12 days after arrival of the veal calves was not associated with the number of BRD treatments performed by the producer, ultrasonographic lung consolidation or weight gain. In this cohort of calves, the proportion of calves treated for BRD by the producer was low 14% (29/209). However, 13% (26/209) of calves included in the study already had ultrasonographic lung consolidation lesions 12 days after their arrival, which was before treatment time, and 27% (56/209) had lung consolidation at D30.

One of the major limitations of this study was the absence of identification of the etiological agent involved. In vivo*,* Tildipirosin was proven efficacious against *Mannheimia hemolytica, Pasteurella multocida* and *Histophilus somni* [11] but evidences of the effectiveness against others agents especially *Mycoplasma bovis* is limited [19]. Moreover, no necropsy was performed. Thus, despites the fact that four dead calves on five had lung consolidation with DEPTH > 3 cm at the last TUS before death and two among this four had been treated for BRD, we cannot concluded on the real cause of death and proved a superiority of TILD (no dead calves) vs control (five dead calves) on mortality. However, since only 2 out of 4 of the dead calves were diagnosed by the owner as having BRD, these results underline the already reported poor sensitivity of owners to diagnose BRD in cattle [20, 21].

The second major limitation of this study was the low apparent BRD clinical and lung consolidation prevalence in this cohort of calves with a proportion of calves treated for BRD by the producer of 14% (29/209) and lung consolidation of 27% (56/209). This may have limited our ability to find a difference between both treatment groups. Indeed, we calculated our estimated sample size based on finding a difference of 15% in calves treated for BRD. With a proportion of 15%, that means that we should have had a proportion of 0% in the TILD to detect a significant difference, which is almost impossible in a fattening unit. Therefore, even if there was a difference, it could have been missed by the low prevalence in both groups.

Concerning the conclusion from TUS, the absence of difference between both groups have to be interpreted with caution because despite the randomisation process on arrival there was an important, but not significant, difference in the proportion of lung consolidation at D12 between the treatment and the control groups (18%; TILD vs 8% PLAC; *P* = 0.06). When we consider the model without the animal already consolidated at D12, the final multivariable model showed that TILD (*P* = 0.04) was positively associated with ADG2 (Additional file 1: Table S2, S3, S4 and S5). So, probably this difference could have impacted the effect of the metaphylactic treatment but most likely it could have limited the ability to find a difference in favour of the treatment group.

One hypothesis that can also explain the absence of effectiveness of TILD in this study is the inadequate time of administration. In the feedlot industry, reduction of morbidity and the negative impact of BRD on ADG is more important when the metaphylactic treatment is given before the morbidity peak [6], which occurs earlier in feedlot (about one week after arrival). In this study, the moment of TILD administration was determined according to the main expected peak of BRD clinical incidence at about three weeks after arrival (diagnosed by the producer or veterinarian) that is similar with what is reported in veal calves in the literature [1]. In this study, using TUS allowed to diagnose lung lesions associated with clinical and subclinical BRD and provided an accurate ante-mortem assessment of lung health [15, 16]. This tool show a sensitivity and a specificity higher than clinical signs and can detect the disease earlier [15, 16]. In this study, during the first 12 days after arrival, the proportion of calves with ultrasonographic lung consolidation increased from 1% (3/209) at D1 to 12% (26/209) at D12 (see more details in the Additional file 1: Table S1). This finding suggests that BRD develops earlier after arrival and remains undetected for a greater or lesser period before detection of clinical signs. This finding emphasizes that the true peak of BRD in veal calf units differs from the apparent, clinical peak of BRD. Moreover, when we consider the model without the animal already sick at D12 (animal with lung consolidation and RS ≥ 5), the final multivariable model showed that TILD (*P* = 0.03) was positively associated with ADG2 (Additional file 1: Table S6 and S7). Thus, the treatment of tildipirosin should probably be efficient as a prophylactic treatment, when animals are not sick yet. With this knowledge, metaphylactic antimicrobial treatment should be given earlier during the pre-weaning period (before D12) or at arrival [6, 9].

Intention-to-treat analyses were used in this clinical trial. In randomised controlled trials (RCT), if there are losses in follow-up, there are three possible approaches to the analysis: comparison of the groups exactly as randomised (ITT), analysis only of individuals who complied completely with the intervention (compliers-only analysis) or analysis of individuals based on the intervention that was received, regardless of whether the individual was initially randomly allocated to that intervention group [17, 18]. The model ITT is considered as the gold standard for statistical analyses in RCT because it has the advantage of taking into account effects that may be related to the intervention and better reflects real life [17]. In this present study, six out of the seven calves were missing data in the PLAC, and the missing data were mainly due to dead calves indicating a beneficial effect of TILD. This beneficial effect would be hidden with the approach of compliers-only analysis where dead calves are excluded from statistical analysis. Unfortunately, there is no clear consensus on how to deal with missing data. As previously described, we used the methods of “imputation of the median (or mean) of the calf’s own group” or “last observation carried forward” [18]. Consequently, as recommended by Wright and Sim [18], and in order to be sure not to under- or overestimate the treatment effect, we also analysed the data with the compliers-only analysis, i.e. by excluding calves with missing data. As for the model ITT, treatment was not significantly associated with BRD treatments, TUS or weight gain (*P* = 0.89).

In this cohort of veal calves, metaphylactic TILD treatment had no effect on ADG during the first month of feeding, second month of feeding or whole pre-weaning period (*P* > 0.05). To the author’s knowledge, this is the first study to assess the effectiveness of tildipirosin on growth performance in veal calves. Indeed, the only previous study that assessed the effectiveness of tildipirosin over the long term was the label study for the homologation of the product as a metaphylactic treatment in feedlots, and only the clinic effectiveness was taken into account [11]. In our study, apparent prevalence of BRD was estimated by the number of calves treated for BRD by the producer, and no differences between the TILD and PLAC groups were observed (*P* > 0.05). The absence of difference in this study compare with the homologation study, could be explained by the relatively low proportion of calves treated for BRD in this cohort of calves (PLAC group: 15%) in comparison to what was expected (25%). In this study, the proportion of ultrasonographic lung consolidation at D30 was the same in the TILD and PLAC groups. In a previous study, cross-bred dairy heifers were first treated with TILD, challenged with *Mannheimia haemolytica* 10 days later and necropsied three days post challenge [20]. Results of this study showed that lung lesions, mainly lung consolidation, observed at necropsy were less extensive in the group treated with TILD than the control group [20]. Explanations for this difference could be that lung lesions were not evaluated in the same way (presence or absence of lung consolidation vs extension of lung consolidation) and that different or multiple BRD pathogens could have been implicated in the present study.

Finally, absence of effectiveness of tildipirosin on weight gain, the number of BRD treatment and lung consolidation in this cohort underlines the fact that metaphylactic treatments must be based on cohort risk assessment. In feedlot cattle and in veal calves, it was shown that low-risk cattle did not benefit from a metaphylactic treatment [6]. Consequently, it is very likely that this fattening unit with such a low BRD prevalence did not benefit from a metaphylactic antimicrobial treatment.

## Conclusion

In this particular study, the metaphylactic treatment of veal calves at D12 after arrival at a fattening unit did not demonstrate any beneficial impact on growth performance. Additionally, it was not associated with a decrease in the proportion of calves treated for BRD or with lung consolidation on TUS. These results should be confirmed in a veal calf population with a greater BRD prevalence (high risk population). They also underline the need to improve the capacity to identify cohorts of cattle with low or high risk of BRD in order to determine which ones could benefit or not of a metaphylactic antimicrobial treatment. It would also be important to evaluate the effectiveness of metaphylactic treatment administered upon arrival of veal calves in the fattening unit rather than D12 as in the current study.

## Additional file

Additional file 1: Table S1. Descriptive data of disease status of the 209 calves randomly assigned to receive a metaphylactic antimicrobial treatment of tildipirosin (TILD) or placebo (PLAC) at D12. **Table S2.** Results from the multivariable logistic regression analysis showing variables that were associated with the presence of lung consolidation at D30, excluding calves already ill at D12 (RS ≥ 5). **Table S3.** Results from the multivariable logistic regression analysis showing variables that were associated with the presence of lung consolidation at D30, excluding calves already ill at D12 ((RS ≥ 5) and consolidated. **Table S4.** Results of the univariable analyses assessing the association between the average daily gain during the 1st month of feeding (ADG1), the 2nd month of feeding (ADG2) and during whole the pre-weaning period (ADG tot), and potential covariates measured during the study. **Table S5.** Results of the multivariable linear regression analysis showing variables that have an impact on average daily gain during the second month of feeding (ADG 2). **Table S6.** Results of the univariable analyses assessing the association between the average daily gain during the 1st month of feeding (ADG1), the 2nd month of feeding (ADG2) and during whole the pre-weaning period (ADG tot), and potential covariates measured during the study. **Table S7.** Results of the multivariable linear regression analysis showing variables that have an impact on average daily gain during the second month of feeding (ADG 2) (DOCX 37 kb)

## Abbreviations

BRD: Bovine respiratory disease
DEPTH: Maximal depth of consolidation
FPT: Failure of passive transfer
PLAC: Placebo
RCT: Randomised controlled trials
RS: Respiratory scoring
SQ: Subcutanously
TILD: Tildipirosin
TUS: Thoracic ultrasonography
