# Supplementary material for: A randomised clinical trial of a metaphylactic treatment with tildipirosin for bovine respiratory disease in veal calves
Source: BMC Vet Res. 2017 Jun 14;13:176. doi: 10.1186/s12917-017-1097-1 (PMC5471741; doi:10.1186/s12917-017-1097-1)
Supplement: Additional file 1: Table S1. — Descriptive data of disease status of the 209 calves randomly assigned to receive a metaphylactic antimicrobial treatment of tildipirosin (TILD) or placebo (PLAC) at D12. Table S2. Results from the multivariable logistic regression analysis showing variables that were associated with the presence of lung consolidation at D30, excluding calves already ill at D12 (RS ≥ 5). Table S3. Results from the multivariable logistic regression analysis showing variables that were associated with the presence of lung consolidation at D30, excluding calves already ill at D12 ((RS ≥ 5) and consolidated. Table S4. Results of the univariable analyses assessing the association between the average daily gain during the 1st month of feeding (ADG1), the 2nd month of feeding (ADG2) and during whole the pre-weaning period (ADG tot), and potential covariates measured during the study. Table S5. Results of the multivariable linear regression analysis showing variables that have an impact on average daily gain during the second month of feeding (ADG 2). Table S6. Results of the univariable analyses assessing the association between the average daily gain during the 1st month of feeding (ADG1), the 2nd month of feeding (ADG2) and during whole the pre-weaning period (ADG tot), and potential covariates measured during the study. Table S7. Results of the multivariable linear regression analysis showing variables that have an impact on average daily gain during the second month of feeding (ADG 2) (DOCX 37 kb) [file 12917_2017_1097_MOESM1_ESM.docx]

|  | **Number of subclinical* calves** | **Number of Clinical* calves** | | **Number of healthy calves** |
| --- | --- | --- | --- | --- |
|  |  | **DEPTH < 3 cm** | **DEPTH ≥ 3 cm** |  |
| **PLAC (n=100)** |  |  |  |  |
| D1  D12  D30 | 1  6  24(5) | 1  0  0 | 0  2(1)  3 | 98  92  68 |
| BRD1 ^a^ (n=6)  BRD2 ^b^ (n=11)  BRD3 ^c^ (n=6)  **TILD (n=109)** | 3  5(2)  2 | 0  1  2(1) | 0  2  0 |  |
| D1  D12  D30 | 2  14(1)  23(6) | 0  1  1 | 0  4(1)  6(3) | 107  90  79 |
| BRD1 (n=6) | 1 | 0 | 1 |  |
| BRD2 (n=6) | 3(2) | 0 | 1 |  |
| BRD3 (n=8) | 3 | 0 | 1 |  |
|  |  |  |  |  |
|  |  |  |  |  |

Table S1 : Descriptive data of disease status of the 209 calves randomly assigned to receive a metaphylactic antimicrobial treatment of tildipirosin (TILD) or placebo (PLAC) at D12

*Subclinical : calves with DEPTH ≥ 3cm but RS < 5

*Clinical : calves with DEPTH < 3cm but RS ≥5 or calves with DEPTH ≥ 3cm and RS ≥5

(number) number of calves that were consolidated or clinical at the previous examination

^a^ BRD 1 = BRD treatments between D1 and D12

^b^ BRD 2=BRD treatments between D12 and D30

^c^ BRD 3= BRD treatments between D30 and the end of the weaning period

PLAC : placebo treated calves; TILD : tildipirosin treated calves

Table S2: Results from the multivariable logistic regression analysis showing variables that were associated with the presence of lung consolidation at D30, excluding calves already ill at D12 (RS ≥ 5).

| **Variables** | | **Coefficient** | **SE** | **OR** | **95% CI** | ***P*-value** |
| --- | --- | --- | --- | --- | --- | --- |
| Intercept |  | -1.91 | 0.35 |  |  | *< 0.0001* |
| Treatment | PLAC | Referent | - | - | - | - |
|  | TILD | 0.22 | 0.34 | 1.2 | 0.6-2.4 | *0.53* |
| DEPTH d12 | <3cm | Referent | - | - | - | - |
|  | ≥3cm | 0.91 | 0.51 | 2.5 | 0.9-6.9 | *0.07* |
| Total solids | >52 g/L | Referent | - | - | - | - |
|  | ≤52 g/L | 0.91 | 0.36 | 2.5 | 1.2-5.1 | *0.012* |

PLAC: placebo treated calves; TILD: tildipirosin treated calves; DEPTH D12: maximal depth of lung consolidation found during thoracic ultrasonography at day 12 after arrival (i.e. time of injection of TILD or PLAC).

Table S3: Results from the multivariable logistic regression analysis showing variables that were associated with the presence of lung consolidation at D30, excluding calves already ill at D12 ((RS ≥ 5) and consolidated.

| **Variables** | | **Coefficient** | **SE** | **OR** | **95% CI** | ***P*-value** |
| --- | --- | --- | --- | --- | --- | --- |
| Intercept |  | -1.91 | 0.38 |  |  | *< 0.0001* |
| Treatment | PLAC | Referent | - | - | - | - |
|  | TILD | 0.16 | 0.36 | 2.6 | 1.2-5.8 | *0.67* |
| Total solids | >52 g/L | Referent | - | - | - | - |
|  | ≤52 g/L | 0.97 | 0.39 | 1.2 | 0.6-2.4 | *0.013* |
|  |  |  |  |  |  |  |
|  |  |  |  |  |  |  |

PLAC: placebo treated calves; TILD: tildipirosin treated calves; DEPTH D12: maximal depth of lung consolidation found during thoracic ultrasonography at day 12 after arrival (i.e. time of injection of TILD or PLAC).

Table S4: Results of the univariable analyses assessing the association between the average daily gain during the 1^st^ month of feeding (ADG1), the 2^nd^ month of feeding (ADG2) and during whole the pre-weaning period (ADG tot), and potential covariates measured during the study.

|  |  | ***ADG 1*** | | | ***ADG 2*** | | | ***ADG tot*** | | |
| --- | --- | --- | --- | --- | --- | --- | --- | --- | --- | --- |
| **Variables** |  | **LSM (lbs/d)** | **SEM** | ***P-*value** | **LSM (lbs/d)** | **SEM** | ***P-*value** | **LSM (lbs/d)** | **SEM** | ***P-*value** |
| Treatment | PLAC | 1.30 | 0.05 | 0.55 | 2.11 | 0.05 | 0.04 | 1.82 | 0.05 | 0.21 |
|  | TILD | 1.26 | 0.05 |  | 2.26 | 0.05 |  | 1.90 | 0.05 |  |
| Sex | Female | 1.39 | 0.13 | 0.38 | 2.17 | 0.12 | 0.54 | 1.92 | 0.09 | 0.98 |
|  | Male | 1.27 | 0.04 |  | 2.26 | 0.03 |  | 1.91 | 0.03 |  |
| DEPTH D1 | ≥ 3 cm | 1.37 | 0.27 | 0.79 | 2.51 | 0.25 | 0.23 | 2.01 | 0.18 | 0.89 |
|  | < 3 cm | 1.28 | 0.03 |  | 2.28 | 0.03 |  | 1.95 | 0.03 |  |
| DEPTH D12 | ≥ 3 cm | 1.21 | 0.11 | 0.28 | 2.31 | 0.09 | 0.86 | 1.90 | 0.07 | 0.92 |
|  | < 3 cm | 1.30 | 0.04 |  | 2.25 | 0.03 |  | 1.91 | 0.03 |  |
| DEPTH D30 | ≥ 3 cm | 1.29 | 0.07 | 0.87 | 2.33 | 0.06 | 0.45 | 1.96 | 0.05 | 0.67 |
|  | < 3 cm | 1.29 | 0.05 |  | 2.27 | 0.04 |  | 1.91 | 0.07 |  |
| Total solids | ≤ 52 g/l | 1.30 | 0.05 | 0.99 | 2.26 | 0.05 | 0.99 | 1.91 | 0.04 | 0.99 |
|  | >52 g/l | 1.30 | 0.05 |  | 2.25 | 0.05 |  | 1.91 | 0.04 |  |
| BRD treatments | 0 | NA |  |  | 2.30 | 0.03 | < 0.01 | 1.91 | 0.03 | < 0.01 |
|  | ≥ 1 | NA |  |  | 1.81 | 0.16 |  | 1.54 | 0.25 |  |

LSM: Least Squares Means; SEM: Standard Error of the Mean; DEPTH D1: maximal depth of lung consolidation found during thoracic ultrasonography at arrival; DEPTH D30: maximal depth of lung consolidation found during thoracic ultrasonography at day 30 after arrival. BRD treatments represent the proportion of calves treated for bovine respiratory disease by the producer during the feeding period. NA: not available. The breed (not presented here) was not significantly associated with ADG1 (*P*=0.30) or ADG2 (*P*=0.26).

Table S5: Results of the multivariable linear regression analysis showing variables that have an impact on average daily gain during the second month of feeding (ADG 2).

| **Variables** |  | **Estimate** | **SE** | ***P*-value** | **LSM (lbs/d)** | **SEM** |
| --- | --- | --- | --- | --- | --- | --- |
|  | Intercept | 1.5448 | 0.08 | <0.0001 |  |  |
| Treatment | PLAC | Referent |  |  | 2.00 | 0.06 |
|  | TILD | 0.15 | 0.08 | 0.04 | 2.15 | 0.06 |
| BRD treatments | 0 | Referent |  |  | 2.25 | 0.04 |
|  | ≥1 | -0.35 | 0.10 | <0.01 | 1.90 | 0.09 |

LSM: Least Squares Means; SEM: Standard Error of the Mean; BRD treatments represent the proportion of calves treated for bovine respiratory disease by the producer during the feeding period.

Table S6: Results of the univariable analyses assessing the association between the average daily gain during the 1^st^ month of feeding (ADG1), the 2^nd^ month of feeding (ADG2) and during whole the pre-weaning period (ADG tot), and potential covariates measured during the study.

|  |  | ***ADG 1*** | | | ***ADG 2*** | | | ***ADG tot*** | | |
| --- | --- | --- | --- | --- | --- | --- | --- | --- | --- | --- |
| **Variables** |  | **LSM (lbs/d)** | **SEM** | ***P-*value** | **LSM (lbs/d)** | **SEM** | ***P-*value** | **LSM (lbs/d)** | **SEM** | ***P-*value** |
| Treatment | PLAC | 1.30 | 0.05 | 0.65 | 2.10 | 0.05 | 0.03 | 1.81 | 0.05 | 0.18 |
|  | TILD | 1.27 | 0.05 |  | 2.26 | 0.05 |  | 1.90 | 0.05 |  |
| Sex | Female | 1.38 | 0.13 | 0.42 | 2.19 | 0.12 | 0.49 | 1.91 | 0.09 | 0.99 |
|  | Male | 1.27 | 0.04 |  | 2.27 | 0.03 |  | 1.91 | 0.03 |  |
| DEPTH D1 | ≥ 3 cm | 1.35 | 0.26 | 0.90 | 2.48 | 0.23 | 0.54 | 2.05 | 0.18 | 0.76 |
|  | < 3 cm | 1.30 | 0.03 |  | 2.28 | 0.03 |  | 1.98 | 0.03 |  |
| DEPTH D12 | ≥ 3 cm | 1.24 | 0.10 | 0.34 | 2.30 | 0.08 | 0.89 | 1.91 | 0.06 | 0.90 |
|  | < 3 cm | 1.32 | 0.04 |  | 2.25 | 0.03 |  | 1.93 | 0.03 |  |
| DEPTH D30 | ≥ 3 cm | 1.28 | 0.07 | 0.81 | 2.32 | 0.06 | 0.62 | 1.93 | 0.05 | 0.72 |
|  | < 3 cm | 1.29 | 0.05 |  | 2.28 | 0.04 |  | 1.90 | 0.07 |  |
| Total solids | ≤ 52 g/l | 1.32 | 0.05 | 0.56 | 2.25 | 0.05 | 0.99 | 1.90 | 0.04 | 0.97 |
|  | >52 g/l | 1.30 | 0.05 |  | 2.25 | 0.05 |  | 1.91 | 0.04 |  |
| BRD treatments | 0 | NA |  |  | 2.27 | 0.03 | < 0.01 | 1.99 | 0.03 | < 0.01 |
|  | ≥ 1 | NA |  |  | 1.77 | 0.18 |  | 1.57 | 0.26 |  |

LSM: Least Squares Means; SEM: Standard Error of the Mean; DEPTH D1: maximal depth of lung consolidation found during thoracic ultrasonography at arrival; DEPTH D30: maximal depth of lung consolidation found during thoracic ultrasonography at day 30 after arrival. BRD treatments represent the proportion of calves treated for bovine respiratory disease by the producer during the feeding period. NA: not available. The breed (not presented here) was not significantly associated with ADG1 (*P*=0.30) or ADG2 (*P*=0.26).

Table S7: Results of the multivariable linear regression analysis showing variables that have an impact on average daily gain during the second month of feeding (ADG 2).

| **Variables** |  | **Estimate** | **SE** | ***P*-value** | **LSM (lbs/d)** | **SEM** |
| --- | --- | --- | --- | --- | --- | --- |
|  | Intercept | 1.5448 | 0.08 | <0.0001 |  |  |
| Treatment | PLAC | Referent |  |  | 1.97 | 0.06 |
|  | TILD | 0.15 | 0.07 | 0.03 | 2.13 | 0.06 |
| BRD treatments | 0 | Referent |  |  | 2.24 | 0.04 |
|  | ≥1 | -0.38 | 0.10 | <0.01 | 1.86 | 0.09 |

LSM: Least Squares Means; SEM: Standard Error of the Mean; BRD treatments represent the proportion of calves treated for bovine respiratory disease by the producer during the feeding period.
